# 

**Published:** 1890-09

**Authors:** 


### INDEX.

### A.

Abdomen, Penetrating wounds of 234.
Abscess of parotids complicating typhoid fever 477.
Academy of Medicine, Formal opening of the new 640.
Acne vulgaris affecting the back 43.
Acute intestinal obstruction, Treatment of 490.
Addison's Disease, A peculiar case of 275.
Address before Southern Surgical and Gynecological Society 643.
Address, Opening, Atlanta Medical College 515.
Address to graduating class Atlanta Medical College 65.
Adenoid vegetations, The present treatment of 402.
Adenoma of the mammary gland 466.
Alexander's operation 566.
Aneurism of the arch of the aorta 477.
Anniversary 312.
Announcement 694.
Antiseptic, Hydronapthol as an 698.
Anus, Artificial, Point of selection for making 469.
Apostoli's method of the treatment of uterine fibroids 632.
Aristol 440.
Army, The Confederate States, To survivors of the medical corps of 188.
Arsenic in skin diseases 202.
Arsenic, The hypodermic use of 549.
Artificial respiration, A new method of 381.
Association, Medical, of Ga. Programme 124.
Association, The American electro-therapeutic 758.

### B.

Bacteriology, Peroxide of hydrogen 642.
Banquet, Annual of Atlanta Society of Medicine 314.
Book reviews 57, 232, 310, 422, 514, 564, 634, 761.
Brain lesions and heat 218.
Bromoform 486.
Bubo, Treatment of 429.

### C.

Calomel and castor oil, Irritation from 415.
Camphoric acid, Therapeutic use of 251.
Cancrum oris, Gangrenous stomatitis, Report of a case of 616.
Cascara sagrada, Therapeutic notes on 335.
Case of remarkable injury with recovery 587.
Case for diagnosis 475.
Cataract operations, Exclusion of light not beneficial in 427.
Cerebral surgery 383.
Chancroid, Scraping out (curetting) 353.
Chancroid, Treatment of by the potassate of sozoiodol 218.
Children in schools, Scientific study of condition of 574.
Chill and fever, Treatment of by potassæ nitras 12.
Chloralamide as a hypnotic for the insane 253.
Chloroform, Fatal after effects of 566.
Chloroform, The administration of by gaslight 428.
Chloroform, The use of in normal labor 86.
Chloroform *vs.* Ether 242.
Circumcision, Operation for 73.
"Clinic," The 697.
Clinic report 55, 179.
Cocaine nitrate in the urinary passages 428.
Coccygodinia 1.
Codeia in diseases of women 237.
Coffee 541.
Collegiate education and the medical profession 380.
Colotomy, Inguinal with report of case 660.
Confederate army and navy, Medical corps of and report of Jos. Jones, M. D. 339.
Congress, International Medical, Tenth 191.
Consumption, Creosote in 485.
Consumption, Koch's cure for 613.
Correspondence, New York letters, etc., 48, 119, 182, 226, 299, 354, 419, 485, 534, 611, 755.
Cough, its relation to intra-nasal disease —.
Cystitis from prostatic hypertrophy, resume of operations 551.
Cystotomy, Perineal *vs.* supra-pubic 550.

### D.

Damages for slander 246.
Defects of skin, bone and widely exposed joints, The covering over of 250.
Diabetes, is it transmissible? 433.
Diabetes mellitus after extirpation of the pancreas 447.
Diphtheria 699.
Diphtheria produced by a disease germ 245.
Diplomas, Bogus 696.
Doctor W. D. Bizzell 376, 575.
Doctor Robert Battey, 376.
Doctor A. W. Calhoun 376.
Doctor W. F. Westmoreland 374, 375.
Doctors, Crowds of, everywhere 426.
Doctors, Southern and their work 495.
Doctors who advertise 392.
Double personality 440.
Drunkenness, The legal and medical aspect of 229.
Dyspepsia, Infectious and its rational treatment by the antiseptic method 539.

### E.

Ear, middle, disease, importance of early treatment 329.
Ectopic Pregnancy, Laparotomy vs. Electricity in 626.
Eczema, A general outline of the treatment 193, 261.
Eczema papulatum venenatum 555.
Editorial 59, 125, 234, 312, 372, 425, 490, 567, 636, 763.
Education, A plea for higher 398.
Electrical execution 497.
Electricity, Preliminary report of post mortem changes in first person executed by 460.

Empyema, Causation and treatment 91.
Endometritis 255.
Entozoa in kidney 55.
Epilepsy, Irregular forms of, with report of a case 631.
Erysipelas 489, 56.
Exhibition, International medico—scientific 190.
Expert testimony 619.
Explanatory 642.
Expression of Placenta by Crede's method 226.
Exsection of shoulder 337.
Eye and ear, suggestions of treatment of 93.

F.

Face presentations 82.
Fad, The latest 764.
Fevers, Continued 579.
Fevers, continued, of the South 621.
Fevers, laws of in relation to cerebral heat centers 218.
Fevers of middle Tennessee and their treatment 620.
Fracture at elbow joint 619.
Fractures, plaster of Paris in recent 280.

G.

Gastro-enterostomy with Senn's approximating plates 243.
Gynecology, plea for conservatism in minor —.

H.

Hemorrhagic Malarial Fever, report of a case 609.
Hemorrhage, Torsion of arteries as a means of arresting 546.
Hip joint, dislocation of 367.
Hospital, The Grady 764.
Hospitals, lying in secrecy, in 427.
Howell's, Dr. case, Concerning 712.
Hydrastinin as an anti-rheumatic 235.
Hydrocele 44.
Hydrocele, treatment of 313.
Hypnotism in its relation to surgery 548.

I.

Ileus, question of the surgical treatment of 386.
Infectious diseases, responsibilities of physicians who attend patients affected with 569.
Influenza, the rationale of 705.
Ingrowing toe nail 494.
Instrument, urethral, exhibition of 470.
Intestinal obstruction, treatment of acute 490.
Items 425, 448, 766, 770, 314, 315, 570, 577, 640, 642.

K.

Koch's lymph 697.
Koch's lymph in lupus 763.
Koch's new treatment for consumption 636.

L.

Labor, erratic pain in 416.
Labor, induction of premature 431.
Labor, modern methods of managing lingering 570.
LaGrippe, diagnosis and treatment of 48.
Laparotomy, report of four cases of 142.
Laparotomies, report of five 456.
Laparotomies, twelve consecutive 321.
Leucorrhœa and blennorrhœa in women 641.
Liver, abscess of 623.
Liver, Experimental contributions to the pathology of 440.

M.

Magazine, The University Medical 699.
Malarial hematuria, "so-called," report of eleven cases of 715.
Mammary gland, adenoma of 466.
Mammary gland, scirrhus of 467.
Mastoiditis in the Negro 478.
Medical Association, Tri-State 426.
Medical Congress, International 425.
Medical Congress, Tenth International 191.
Medical education 425.
Medical progress, help and hindrance to 541.
Membrana tympani, rupture of by injection of warm water into the auditory canal, etc. 707.
Menstrual derangement 308.
Midwifery, application of antiseptic methods 553.
Myomotomy in a pregnant woman 554.

N.

Naso-pharynx, general remarks on some of the pathological conditions of 452.
Nasal reflexes 257.
Neurosis, a case of 619.
Nitrate of cocaine in the Urinary passages 428.

O.

Obituaries, Drs. Bizzell, Westmoreland and Word 320.
Obstetrics and gynecology 14.
Ophthalmitis, sympathetic, pathogenesis and new treatment of 442.
Organic heart disease, should set a limit to life in with caution 478.
Osteosarcoma of orbit 469.
Osteosarcoma, report of *post mortem* in a case of 462,
Ovarian tumors, two cases of rare 179.
Ovariotomies, report of two difficult 137.
Ovary and tube, partial resection of diseased 430.

P.

Pancreas, diabetes mellitus after extirpation of 447.
Paracentesis abdominis, aseptic apparatus for 73.
Parotitis complicating typhoid fever, fatality in 477.
Pelvic inflammation, how should we treat our cases of 605.
Pericarditis, fatal, three cases of 369.
Peritonitis, local and general treatment of 209.
Peritonitis, treatment of 557.
Peroxide of Hydrogen, the necessary 435.
Personals 496.
Pharmacology and Therapeutics 700.
Phenacetin as an anti-rheumatic 238.
Phenacetin in Whooping cough 239.
Phthisis, creosote in treatment of 674.
Phthisis, Night sweats of, potassium tellurate in 575.
Phthisis, Prof. Flint's doctrine of the self-limitation of 523.
Placenta, a case of adherent 18.
Placenta, expression of by Crede's method 226.

Pleurisy, acute 611.
Poisoning by anilides 62.
Post-partum hemorrhage, treatment of 247.
Potassii Nitras 54, 231.
Preachers, patent medicines and doctors' bills 695.
Pregnancy, examination for uræmic symptoms in, imperative 415.
Pregnancy, tubal, two cases of 551.
Prizes, the Mattison 698.
Puerperal convulsions, a case of, four days after delivery 532.
Puerperal convulsions, report of a case 409.
Pueperal Convulsions, veratrum viride in 413.
Purpura hemorrhagica 612.
Pyosalpinx, treatment of 59.
Pyoxtaninum aureum and cæruleum in soft chancres and syphilitic affections 615.

Q.

Quinine, the abuse of 395.
Quinine, the true value of, in continued fevers 434.

R.

Radius, fracture of lower end of 699.
Rectum, scirrhus of in child of 13 years 417.
Reflex pain, obscure cases illustrating the necessity of vaginal examination 44.
Relapsing fever? was it 602.
Resolutions of respect 765.
Resolutions of respect to the memory of Dr. Willis F. Westmoreland 375.
Respiration, artificial, a new method of 381.
Retinal detachment, treatment of, etc. 209.

S.

St. Bartholomew's Mission 429.
Salol 438.
Salpingitis, chronic catarrhal, treatment of by electricity 29.
Sarcoma, fibro, advisability of removal 147.
Scirrhus of rectum in child of 13 417.
Scirrhus of rectum in child of 13, diagnosis confirmed 468.
Scleroderma, generalized, a case of 333.
Secretion of bile, action of olive oil on 248.
Selection —.
Shoulder, dislocation of 369.
Shoulder, exsection of 337.
Skin diseases, one hundred consecutive cases of I. 684, II. 725.
Soap and water, use and abuse of 254.
Society Reports: Ga. State 170; Pittsburgh Obstetrical 96; Philadelphia Obstetrical 29; Allegheny county 101, 151, 364, 470, 740; Atlanta 43, 112, 290, 481, 554, 557, 751; Gynecological and Obstetrical, Baltimore 729; Tri-State Medical 616; Mississippi Valley 539; Richmond 462, 474, 412; Tenn. State 157.
Soda, silicate in surgery, some new methods of use of 623.
Somnal, observations on 432.
Somnal, the hypnotic, notes upon 701.
Southern Surgical and Gynecological Association (Ed.) 639.
Spectacles, a plea for the more extended use of as a therapeutic measure 606.
Speech and locomotion absent in a child three and a half years of age 412.
Stab-wounds of abdomen, cases of penetrating, laparotomy, results 515.
Sterilization of water 383.
Stricture, treatment of organic of male urethra 552.
Sulphonal 236.
Sulphonal, the action of 240.
Summer complaints 241.
Surgeon and sentiment 128.
Surgery, Cerebral 383.
Surgery, Pelvic, motive and method of 579.
Suspicious growths, early removal of advocated 466.
Symphisis pubis, wiring the separated 547.
Syphilis 677.
Syphilis, electrical cataphoresis in treatment of 573.
Syphilitic brain lesions, two case of 73.

T.

Theory, A 283.
Thermometer, Clinical, "duplex instanter" 446.
Tonsils, Hypertrophy of 628.
Tracheotomy for foreign bodies in the air passages 449. Discussion of 283.
Tribute to the memory of Dr. W. D. Bizzell, 376.
Typhoid fever, a case of 272.
Typhoid fever, loss of speech in 366.
Typhoid fever, treatment of 50.

U.

Umbilical cord around neck 53.
Umbilical hernia, radical cure of 640.
Uræmia, pilocarpine in 415.
Uræmic symptoms, examination for in pregnancy, imperative 415.
Ureter, a third emptying into the urethra 640.
Urethra, the female, a source of trouble often overlooked in our gynecological practice 385.
Urethral stricture and its complications 632.
Urinary passages, nitrate of cocaine in 428.
Uterine fibroids, notes on Apostoli's treatment of 632.
Uterine fibroma 624.
Uterine treatment, the abuse of 377.
Uterus, mechanical obstruction in diseases of 541.

V.

Varicose veins 566.
Veratrum viride in puerperal convulsions 413.
Volvulus, case of suspected 364.

W.

Whooping cough, bromoform in 574.

